# Nebulized antibiotics in mechanically ventilated patients: a challenge for translational research from technology to clinical care

**DOI:** 10.1186/s13613-017-0301-6

**Published:** 2017-08-01

**Authors:** Stephan Ehrmann, Jean Chastre, Patrice Diot, Qin Lu

**Affiliations:** 1 Médecine Intensive Réanimation, Réseau CRICS-TRIGGERSEP, Centre Hospitalier Régional et Universitaire de Tours, Tours, France; 2 Centre d’études des Pathologies Respiratoire, INSERM U1100, Faculté de Médecine de Tours, Université François Rabelais de Tours, Tours, France; 3 Service de Réanimation Médicale, Institut de Cardiologie, Assistance Publique-Hôpitaux de Paris, Pitié-Salpêtrière Hospital, UPMC (University Pierre and Marie Curie) Paris-6, Paris, France; 4 Pneumologie, Centre Hospitalier Régional et Universitaire de Tours, Tours, France; 5 Multidisciplinary Critical Care Unit, Department of Anesthesiology and Critical Care Medicine, Assistance Publique-Hôpitaux de Paris, Pitié-Salpêtrière Hospital, UPMC (University Pierre and Marie Curie) Paris-6, Paris, France

**Keywords:** Nebulizers and vaporizers (MeSH), Pneumonia, ventilator-associated (MeSH), Colistin (MeSH), Amikacin (MeSH)

## Abstract

Nebulized antibiotic therapy directly targets airways and lung parenchyma resulting in high local concentrations and potentially lower systemic toxicities. Experimental and clinical studies have provided evidence for elevated lung concentrations and rapid bacterial killing following the administration of nebulized antibiotics during mechanical ventilation. Delivery of high concentrations of antibiotics to infected lung regions is the key to achieving efficient nebulized antibiotic therapy. However, current non-standardized clinical practice, the difficulties with implementing optimal nebulization techniques and the lack of robust clinical data have limited its widespread adoption. The present review summarizes the techniques and clinical constraints for optimal delivery of nebulized antibiotics to lung parenchyma during invasive mechanical ventilation. Pulmonary pharmacokinetics and pharmacodynamics of nebulized antibiotic therapy to treat ventilator-associated pneumonia are discussed and put into perspective. Experimental and clinical pharmacokinetics and pharmacodynamics support the use of nebulized antibiotics. However, its clinical benefits compared to intravenous therapy remain to be proved. Future investigations should focus on continuous improvement of nebulization practices and techniques. Before expanding its clinical use, careful design of large phase III randomized trials implementing adequate therapeutic strategies in targeted populations is required to demonstrate the clinical effectiveness of nebulized antibiotics in terms of patient outcomes and reduction in the emergence of antibiotic resistance.

## Background

Effective antimicrobial therapy requires adequate drug concentrations at the site of the infection. This is often not possible when using intravenous therapy among intensive care unit (ICU) patients who require mechanical ventilation due to altered pharmacokinetics and poor lung tissue penetration of many antimicrobial agents [1, 2]. Outcome is often suboptimal, with clinical response rates of lower than 60%, even for antibiotic-susceptible bacterial pneumonia [3]. The situation is particularly challenging when bacteria with a minimum inhibitory concentration (MIC) close to the resistance breakpoint are involved [4]. Raising the systemic antibiotic dose leads to increased toxicity. Nebulized antibiotic therapy directly targets airways and lung parenchyma, thereby resulting in increased local concentrations and hence potentially improving efficacy and minimizing toxicities [5, 6]. For patients suffering from cystic fibrosis, for whom maintaining intravenous access can be challenging and who frequently develop lung infections with bacteria exhibiting reduced antibiotic sensitivity, these theoretical advantages have led to large-scale clinical implementation of nebulized antibiotic therapy and improved patient-centered outcomes [7, 8]. In the setting of critically ill patients undergoing mechanical ventilation, despite similar theoretical advantages to treat ventilator-associated pneumonia (VAP), practical issues regarding the use of nebulized drugs and an overall lack of robust clinical data have limited their widespread adoption.

The present review summarizes current practical constraints for optimal delivery of nebulized antibiotics to the lung parenchyma during invasive mechanical ventilation, and the resulting pharmacokinetics and pharmacodynamics. Current clinical practice is put into perspective with evidence that has become available from recent clinical studies so as to provide a better understanding of the relevance of future phase III trials.

## Practical constraints to optimizing nebulized antibiotic delivery during mechanical ventilation

Delivery of high concentrations of antibiotics to infected lung regions is the key to achieving efficient nebulized antibiotic therapy. The antibiotic dose placed in the nebulizer should take into account the significant extrapulmonary drug deposition (i.e., the residual antibiotic volume remaining in the nebulizer chamber, ventilator circuit and endotracheal tube deposition, and exhaled particles). Poor implementation may result in extrapulmonary deposition as high as 97% [9]. Key practical factors need to be taken into account to optimize delivery.

### Particle size

The optimal mass median aerodynamic diameter that allows for distal lung deposition ranges from 0.5 to 3 µm [10]. Particles larger than 5 µm are subject to pronounced deposition in the ventilator circuit and the large airways.

### Nebulizer

Table 1 displays advantages and drawbacks of available nebulizers. Jet nebulizers appear to be less efficient than ultrasonic and vibrating mesh nebulizers for antibiotic delivery [11, 12]. The large residual volume of medication remaining in the chamber at the end of nebulization, as well as high-speed turbulent flow due to the gas driving the nebulizer, underlies these results. Vibrating mesh nebulizers appear to be advantageous compared to ultrasonic devices due to a smaller residual volume and because the temperature of the medication does not increase significantly during nebulization [13].

**Table 1:** Advantages and disadvantages of three types of nebulizers

	Jet nebulizer	Ultrasonic nebulizer	Vibrating mesh nebulizer
Mechanism of aerosol generation	Compressed gas and Venturi effect	High-frequency drug solution agitation by a piezoelectric crystal	High-frequency mesh vibrations pumping the drug solution trough tapered holes
Residual volume	Large	Medium	Small
Medication restriction	None	Degradation of heat-sensitive drugs	Highly concentrated or viscous solutions may cause damage to the nebulizer
Ergonomics	Not portable, need of compressed gas Loud Disposable Potential interference with the ventilator	Bulky Silent Need for decontamination No interference with the ventilator	Portable, small size Silent Disposable No interference with the ventilator

The particle sizes generated depend on each individual nebulizer model rather than the nebulizer type, and they are substantially impacted by the measurement conditions (e.g., temperature and humidity). For example, some specific jet nebulizers may deliver large particles (>5 µm for proximal targeting), whereas others deliver nanoparticles. All nebulizers available for clinical use produce sufficient droplets in the 1–5 µm size range of for pulmonary delivery during mechanical ventilation

### Drug concentration

Medication dilution and the nebulizer fill volume influence particle size and drug delivery. For a given dose, a larger fill volume with a diluted solution can overcome the residual volume issues mentioned above [14]. Nevertheless, dilution increases the duration of the nebulization, and as a result, issues with antibiotic stability may arise. For example, solubilized colistimethate sodium (CMS) is not stable and its antimicrobial efficacy decreases over time [15]. Conversely, a highly concentrated or viscous solution increases the particle size, potentially decreasing lung deposition [16]; it may also induce obstruction or damage when used with a vibrating mesh nebulizer.

### Nebulizer position

**Fig. 1:**
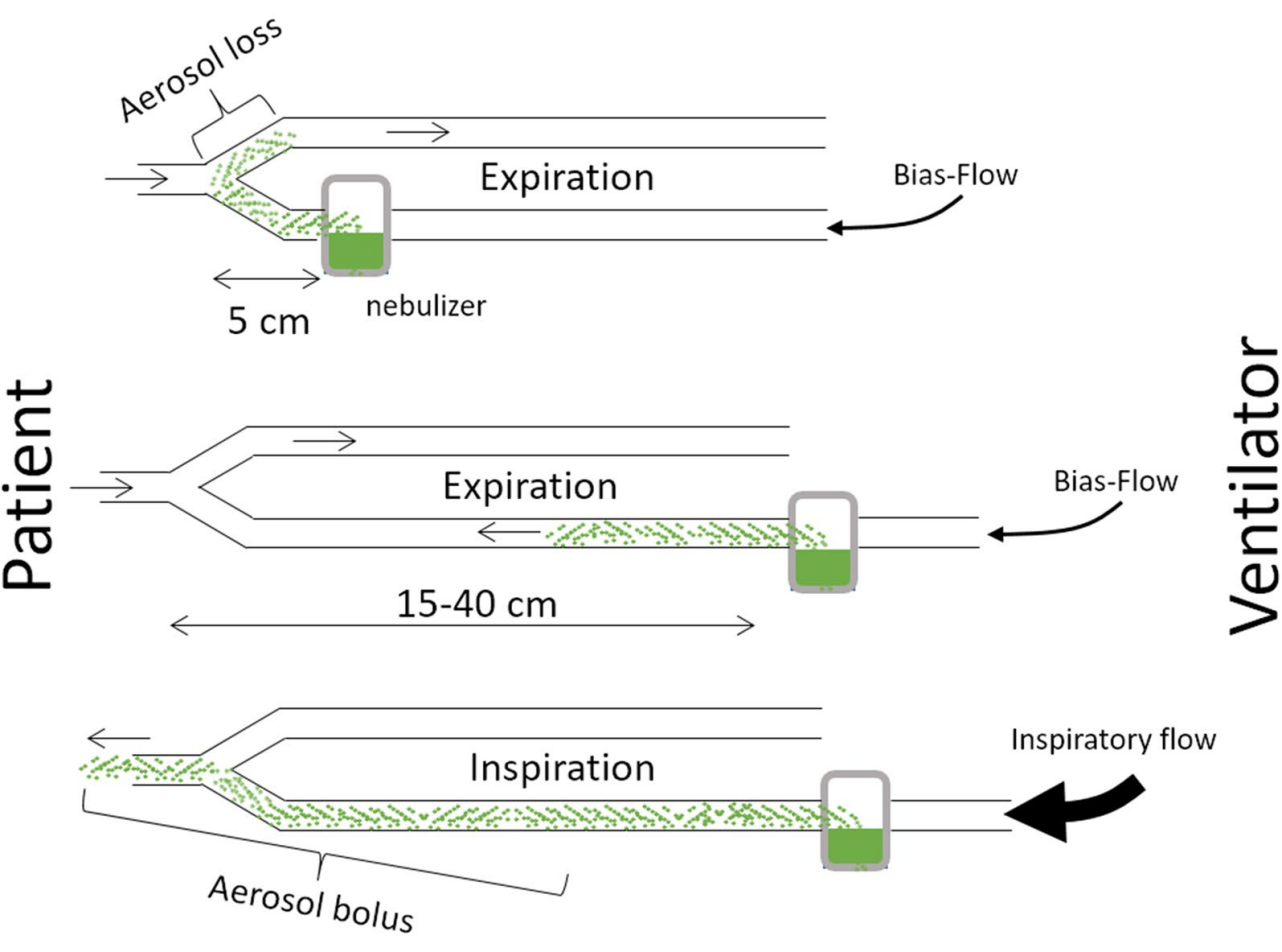
Influence of the nebulizer position on aerosol losses during expiration. Nebulizer positioning upstream in the inspiratory limb enables the latter to act as a spacer/reservoir, thereby storing aerosol during expiration for an aerosol bolus delivery at the next insufflation

A nebulizer operating continuously during both insufflation and expiration should be placed in the inspiratory limb, 15–40 cm upstream of the *Y*-piece [11, 17]. The optimal distance from the *Y*-piece depends on bias flow and the circuit section. Indeed, the bias flow flushes aerosol into the expiratory limb during expiration, inducing aerosol loss (Fig. 1) [11, 18]. Breath-actuated nebulization, which occurs only during insufflation, offers theoretical advantages in light of the reduced expiratory loss. A nebulizer placement closer to the *Y*-piece may hence be an option [18]. Moreover, breath-actuated jet nebulizers enable tidal volume control, as opposed to using driving gas external to the ventilator, which is a practice that should be avoided [19]. Breath-synchronized vibrating mesh nebulizers are currently undergoing investigation, and they may overcome the poor synchronization observed with current jet systems [19]. However, breath synchronization comes at the cost of increased treatment durations [20], and direct comparison with continuous nebulization performed under optimal conditions requires further studies.

### Circuit humidification and filter

Humidified gas increases the size of the aerosol particles through hygroscopic water absorption. Decreased efficiency has been demonstrated to occur in heated and humidified as compared to dry ventilator circuits [21, 22]. As heat and moisture exchangers present a complete barrier to aerosol delivery, they should be removed during nebulization, thus interrupting passive humidification. When using an active heated humidifier, switching it off during nebulization may be an option. However, the decrease in humidity and temperature may be slow and the benefit on nebulization is questionable [23]. An effective way to reduce humidity during nebulization is to use a dedicated dry ventilator circuit during nebulization. Although nebulization by itself exerts some form of humidification, caution should be taken when nebulization lasts more than 1 h to avoid damage to the ciliated epithelium and endotracheal tube occlusion [24].

### Ventilator settings

Theoretically, a laminar low inspiratory flow is required to promote distal lung aerosol deposition [25]. Ventilator settings that enhance nebulization efficacy include a low respiratory frequency, low inspiratory flow and increased inspiratory time [14, 26]. Volume-controlled ventilation with constant low inspiratory flow increases efficacy compared to pressure-controlled ventilation (high peak flow followed by deceleration) [17, 27]. An end inspiratory pause may facilitate the settling of aerosol particles in the lung [26]. Complete ventilator synchrony may reduce turbulence and improve efficacy. Tolerance of such specific ventilator settings in patients who are awake may be poor and the benefit-to-risk ratio of temporary sedation during nebulization should be evaluated on a case-by-case basis.

These practical constraints present a substantial hurdle in regard to performing clinical trials and ultimately for the feasibility of large-scale nebulized antibiotic therapy in daily clinical routine.

## Pharmacokinetics and pharmacodynamics

The efficacy of antibiotic therapy depends on pharmacokinetic and pharmacodynamic criteria. Antibiotics with poor diffusion through biological membranes are appropriate candidates for nebulization, as intravenous infusion results in low lung concentrations. Indeed, low concentrations of tobramycin were detected in lung epithelial lining fluids (ELF) following intravenous infusion of 7–10 mg/kg of this drug [28]. Even when the intravenous amikacin dose is increased to 25–30 mg/kg, the target level is rarely reached [2, 29]. Similarly, in regard to polymyxins, several studies have shown a lack of lung tissue penetration of colistin when CMS was administered intravenously [30, 31]. This issue remains controversial, however. Markou et al. [32] were able to detect colistin in lung ELF in two patients after 4 and 12 days, respectively, of intravenous CMS. The pharmacological complexity of CMS and colistin should be pointed out. CMS under in vivo physiological conditions is hydrolyzed into 32 different compounds, among which colistin A and colistin B represent 85% of the mixture and exert most of the antibacterial activity. CMS itself has no antibacterial activity. It is hence difficult to characterize the kinetics of the formation and absorption of colistin after CMS infusion or nebulization.

From a pharmacodynamic point of view, aminoglycosides and colistin are concentration-dependent antibiotics with a post-antibiotic effect. They are particularly suitable for nebulization as high lung concentrations can be expected and only 1 to 3 daily administrations are required. Time-dependent antibiotics, such as β-lactams or glycopeptides, require drug concentrations to be maintained above the MIC throughout the dosing interval. Continuous or closely repeated administration is hence required [33, 34], which could limit the clinical feasibility of nebulized delivery of such drugs.

Nephrotoxicity associated with CMS and aminoglycosides after intravenous infusion represents an additional rationale for their nebulized delivery. Given the lack of a proven benefit and increased nephrotoxicity, a recent Cochrane systematic review discouraged the use of intravenous aminoglycoside in combination with β-lactam antibiotics for treating sepsis [35]. Conversely, systemic uptake, albeit not negligible [36, 37], is limited after nebulization. Therefore, nebulization of hydrophilic drugs such as aminoglycosides, polymyxins and glycopeptides presents a favorable pharmacokinetic profile, thus potentially limiting nephrotoxicity [1].

**Fig. 2:**
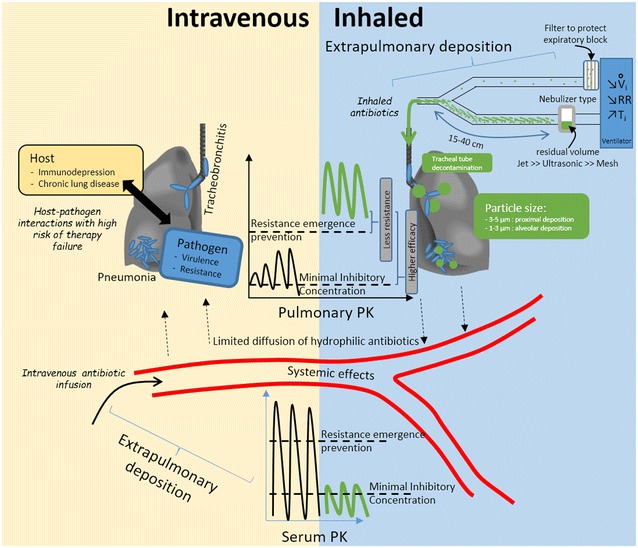
Differences between intravenous and nebulized antibiotic therapy. Intravenous infusion (*yellow panel*, *left bottom corner*) leads to high extrapulmonary concentrations and potential toxicities. Diffusion to the lung is limited and resulting concentrations that may not exceed minimal inhibitory concentration can lead to treatment failure in challenging host–pathogen combinations. Nebulized delivery (*blue panel*, *right top corner*), implementing an optimized technique (detailed in Table 3) results in higher pulmonary concentrations that are above the resistance emergence prevention threshold, thus reducing the likelihood of resistant strain selection. These concentrations are well above the minimal inhibitory concentration, thus resulting in improved efficacy of concentration-dependent antibiotics, even with difficult-to-treat pathogens; systemic side effects may be reduced. Nebulization requires carful implementation so as to avoid potential respiratory side effects. *PK* pharmacokinetics, *V*_i_ inspiratory flow, *RR* respiratory rate, *T*_i_ inspiratory time

These theoretical favorable pharmacokinetic and pharmacodynamic profiles, summarized in Fig. 2, have been documented in experimental and clinical studies involving optimized nebulization techniques for amikacin and colistin.

### Experimental evidence of favorable pulmonary pharmacokinetics

The efficacy of 45 mg/kg/24 h nebulized amikacin has been studied in ventilated piglets with pneumonia due to *Escherichia coli*. The amikacin concentrations measured in the infected lung parenchyma were significantly higher than the MIC and 3–30 times higher than after intravenous infusion [5]. No pulmonary or systemic accumulation was observed over three days in piglets with normal kidneys [5, 38]. The efficacy of 8 mg/kg/12 h nebulized CMS has been studied in ventilated piglets with pneumonia due to *Pseudomonas aeruginosa*. Peak concentrations in the infected lung parenchyma were significantly higher than the MIC [30]. In an experimental sheep model, no colistin was quantifiable in the ELF after intravenous infusion of CMS, whereas high ELF colistin concentrations were measured after nebulization [39]. Improved bacterial killing after nebulization was also observed in these animal studies compared to intravenous administration of the drug [5, 30, 39].

These experimental studies, documenting high lung parenchymal antibiotic concentrations after nebulization of high doses and implementing optimized techniques, laid the foundations for clinical pharmacokinetic studies.

### Clinical evidence of favorable pulmonary pharmacokinetics

It was recently shown that in patients with healthy lungs, 10–15% of the nebulizer charge is deposited in the lungs [17]. Such a level of delivery is compatible with high drug concentrations in the lung, although a substantial level of heterogeneity within the lung and a predominantly proximal deposition pattern were observed.

In patients with VAP, nebulized amikacin (400 mg/12 h) achieved median lung ELF concentrations 100 times higher than the maximum serum concentration [40]. Similarly, Niederman et al. [41] measured very high amikacin concentrations in the tracheal aspirate of patients suffering from VAP after nebulized amikacin (400 mg/12 h). In both studies [40, 41], a vibrating mesh nebulizer synchronized with inspiration was used [42]; patients were ventilated in pressure-controlled or volume-controlled mode, and heated humidification was performed during the nebulization. Despite the use of an only partially optimized nebulization technique and a relatively low amikacin dose compared to experimental data, it is likely that high amounts of amikacin were delivered to the lungs given the specific synchronized device that was used [42]. Systemic absorption remained low. Another group tested a combination nebulization of low-dose amikacin (100–500 mg) and fosfomycin (40–200 mg) in patients with VAP [43]. Although the continuously operating vibrating mesh nebulizer was optimally placed in the ventilator circuit, the ventilator settings were not controlled. Again, very high antibiotic concentrations were measured in bronchial secretions, even in patients receiving the lowest amikacin doses (concentrations above 5000 µg/mL, amounting to 40 times the serum peak concentration). Using higher amikacin doses with a different nebulizer in patients with VAP, Peticollin et al. [37] showed that nebulized doses of up to 60 mg/kg may be safe as they were associated with serum concentrations that were lower than those observed after intravenous infusion of a standard dose. Inter- and intra-patient pharmacokinetic variabilities of serum concentrations were very high in this study. Similarly, high inter-patient variations of amikacin concentrations were observed at the lung level with a factor of 100 in ELF and bronchial secretions [40].

In patients with VAP or ventilator-associated tracheobronchitis (VAT) who were administered 1 million international units (MIU, i.e., 80 mg) of CMS via a vibrating mesh nebulizer under optimal conditions every 8 h, peak ELF concentrations were high but then dropped below the sensitivity breakpoint at 4 h, thus indicating that this dose may not be optimal for treating pneumonia [44] (Table 2). Nebulization of CMS at a single dose of 2 MIU, implementing the same optimal nebulization technique, has been reported to yield significantly higher ELF colistin concentrations than after intravenous administration [45]. Steady-state plasma concentrations of colistin, indirectly reflecting alveolar deposition, were significantly higher in studies evaluating high doses of nebulized CMS (4–5 MIU/8 h) [46, 47] compared to 2 MIU/8 h [45]. Systemic exposure of CMS and colistin was significantly lower after nebulization compared to intravenous infusion [6, 45], indicating a reduced risk of nephrotoxicity [47]. The various methods used to measure deposition and the concentration of nebulized antibiotics at the site of infection have several technical limitations. The measurement of ELF concentrations of antibiotics could be skewed by contamination as a result of lysis of ELF cells and technical constraints of bronchoalveolar lavage [48], as well as reliable assessment of the unbound drug concentration in the lung. Further development and research, including use of micro-dialysis, are required to better characterize local antibiotic concentrations [49]. Lastly, the inter-subject variability of pharmacokinetics may not have been fully captured given the limited sample size of currently available studies.

**Table 2:** Colistin ELF and plasma concentrations after nebulization with different doses

Study	Athanassa [44] (*n* = 20)^a^	Boisson [45] (*n* = 12)^a^	Bihan [46] (*n* = 1)^b^	Lu [6] (*n* = 16)^b^
Nebulized dose	1 MIU	2 MIU	4 MIU	5 MIU
Nebulizer	Vibrating mesh nebulizer, continuous delivery, optimized conditions			
Colistin assay	HPLC	LC–MS/MS	LC–MS/MS	HPLC
VAP/VAT	VAT	VAP	VAP	VAP
Lung ELFmax (mg/L)	6.73 (4.8–10.1)	1137	NA	NA
Lung ELFmin (mg/L)	2.0 (1.0–3.8)	9.53	NA	NA
Plasma Cmax (mg/L)	1.6 (1.5–1.9)	0.73	2.9	2.2 ± 1.3
Plasma Cmin (mg/L)	0.3 (0.3–0.5)	0.15	2.4	1.4 ± 0.9

Data are presented as mean ± SD, medians (25–75% interquartile) or maximum and minimum values

*HPLC* High-performance liquid chromatography, *VAP* ventilator-associated pneumonia; *VAT* ventilator-associated tracheobronchitis, *ELF* epithelial lining fluid, *LC–MS/MS* liquid chromatography–tandem mass spectrometry, *Cmax* maximum plasma concentration, *Cmin* minimum plasma concentration

^a^ Blood sampled after the first dose; ^b^ blood sample performed at steady-state

## Clinical efficacy

One randomized controlled trial studied the efficacy of nebulized antibiotic therapy only to treat VAP caused by *P. aeruginosa* as compared to intravenous therapy [34]. Forty patients were allocated to receive either nebulized ceftazidime (15 mg/kg every 3 h) and amikacin (25 mg/kg once per day) or intravenous ceftazidime (90 mg/kg daily continuous infusion) and amikacin (15 mg/kg once per day). Antibiotics were delivered implementing strict optimized nebulization practice via a vibrating mesh nebulizer (a continuous delivery system), ventilator settings were optimized and the humidifier was switched off during the nebulization. In this phase II study, patients who only received nebulized antibiotics had a 70% cure rate compared to 55% for those receiving only intravenous antibiotics. The difference was not statistically significant, however. Interestingly, effective therapy was also observed in patients infected by bacteria with intermediate susceptibility to the nebulized antibiotics. Furthermore, in patients for whom the treatment failed, new or persistent bacterial growth was caused exclusively by susceptible strains in the nebulized group, whereas 50% of the recurrent strains had become intermediately or fully resistant in the intravenous group. High antibiotic lung concentrations well above the MIC and higher than the concentration preventing the emergence of resistance may explain these results (Fig. 2). The median peak plasma concentration after amikacin nebulization was 8.9 mg/L, thus reflecting significant systemic diffusion, with 25% of patients presenting a trough amikacin concentration above 5.9 µg/mL. It is difficult, however, to translate this exclusively nebulized antibiotic therapy into routine practice, as ceftazidime nebulization every 3 h may be considered cumbersome and outweigh potential benefits.

Testing another clinical strategy, Palmer et al. [50] assessed the effects of nebulized antibiotics as an adjunctive therapy to intravenous antibiotics in patients with VAT and/or VAP [51]. Patients with Gram-positive bacteria were treated with vancomycin at 120 mg/8 h and those with Gram-negative organisms were treated with gentamicin at 80 mg/8 h or amikacin at 400 mg/8 h. Antibiotics were delivered via a breath-actuated jet nebulizer with active humidification turned off. Adjunction of nebulized antibiotics to systemic therapy rapidly sterilized bronchial secretions and decreased the VAT/VAP incidence, thus revealing a favorable prophylactic effect on the transition from VAT to VAP and a curative effect for patients with VAP. Nebulization was associated with a faster resolution of signs of infection and weaning, as well as reduced use of systemic antibiotics. Similar to the work by Lu et al. [34], the emergence of drug-resistant bacteria was reduced for patients receiving nebulized antibiotics [51]. Kollef et al. [52] tested a strategy of low-dose nebulized amikacin (300 mg/12 h) combined with fosfomycin (120 mg/12 h) as adjuncts to intravenous antibiotics in patients with VAP. Whereas no significant effect on clinical outcomes was observed, nebulized antibiotics were associated with a faster sterilization of bronchial secretions and again a significantly reduced emergence of drug-resistant bacteria.

The systemic antibiotic sparing effect of nebulized antibiotics was also observed in the earlier mentioned work by Niederman et al. [41]. For 69 patients with VAP, nebulized amikacin (400 mg/12 h or/24 h) reduced the number of systemic antibiotics per patient per day at the end of the 14-day therapy. However, no benefit in terms of clinical responses was observed, and this may have been due to a very high cure rate in the control group.

In these studies, various doses of nebulized amikacin were used (twice as many high-dose versus low-dose studies) and aerosols were delivered with different nebulizers although close attention was often paid to optimize the nebulization technique. Nebulized amikacin allowed for effective treatment of bronchial and parenchymal infections, even when involving bacteria with high MIC, and it consistently reduced the emergence of resistant bacteria. Clinical cure, evaluated as a secondary endpoint in these trials, depends in part on the therapeutic efficacy in the control group. Further clinical phase III studies are required to prove the clinical benefit of nebulized amikacin.

As colistin is the most frequently nebulized antibiotic in ICUs, there is some information in regard to clinical bedside safety and efficacy as well as large retrospective databases. However, only a small number of prospective controlled studies have evaluated nebulized colistin in mechanically ventilated patients implementing optimized technique. High-dose nebulized CMS (5 MIU/8 h with strict optimized technique) has been evaluated in patients with VAP caused by multidrug-resistant (MDR) *P. aeruginosa* and *Acinetobacter baumannii* [6]. Patients with VAP caused by sensitive strains and treated with standard intravenous antibiotics served as controls. The clinical cure rate was 66% in the sensitive strain group and 67% in MDR strain group. By testing a nearly exclusively nebulized therapy strategy (a minority of patients received an intravenous aminoglycoside complement), this study very much indicates that nebulization of high-dose CMS may be effective to treat MDR bacterial VAP. Conversely, in patients suffering from VAP primarily due to sensitive bacteria, a randomized trial testing nebulized colistin as an adjunctive therapy to intravenous antibiotics appeared to yield negative results [53]. A recent randomized trial evaluated high doses of nebulized colistin using an optimized technique either as adjunct to intravenous antibiotics for VAP due to sensitive bacteria or as an exclusively nebulized therapy in case of MDR bacteria. Patients in the nebulized group had a significantly lower incidence of acute renal failure, a higher level of oxygenation and a shortened time to bacterial eradication than those in the control group receiving intravenous colistin [54], although the overall clinical cure rate was not significantly different. The fact that patients infected with MDR bacteria and treated exclusively with nebulized colistin had similar outcomes as patients infected with sensitive bacteria who were treated with intravenous antibiotics in addition to nebulized colistin can be considered to be an encouraging result [54].

Three groups of investigators used databases with information regarding patients suffering from colistin-only susceptible bacterial VAP to evaluate whether nebulized CMS as adjunct to intravenous CMS is beneficial. One study observed no additional benefit of combined nebulized and intravenous CMS therapy [55], whereas the two others observed a higher cure rate compared to intravenous therapy alone [56, 57]. Furthermore, clinical use of intravenous colistin is still a matter of debate [58, 59].

Meta-analyses of the clinical studies have yielded conflicting results and further clinical evidence from randomized trials is required, while more extensive evaluation of renal toxicity related to the administration of high dose of CMS or amikacin intravenously or by nebulization is also needed [60–65]. The currently available evidence cannot be considered to be sufficient for implementation of nebulized antibiotics as a straightforward therapeutic option.

Aside from investigating curative nebulized antibiotics to treat patients suffering from VAT and/or VAP, some authors have also tested nebulized colistin, ceftazidime or aminoglycosides for prophylaxis in intubated patients. Two small-sized studies obtained positive results with such a preemptive nebulized therapy in terms of the VAP frequency, and they also observed no significant change in the bacterial antibiotic sensitivity pattern [66, 67]. Further studies are required to assess this benefit as well as the risk of antibiotic resistance selection pressure.

## Current practice

The spread of MDR associated with the favorable data outlined above led to implementation of nebulized antibiotic therapy in the clinical setting despite the lack of large-scale patient-centered evidence. Among 816 international intensivists surveyed electronically, one-third reported that they usually or frequently nebulize colistin [68]. In an observational study in 80 ICUs, every fifth intubated patient received an aerosol, and 5% involved nebulized antibiotics [69]. Nebulized antibiotics (80% colistin) were delivered to 1% of the ICU patients in 17% of the study centers. A subsequent international survey that specifically investigated the use of nebulized antibiotics in ICUs highlighted very heterogeneous indications ranging from prophylactic or empirical therapy to documented lung infections in immunocompetent and immunocompromised patients, for VAT and VAP [70]. The use of jet nebulizers appears to be predominant, and practical implementations were far from the optimized efficacy conditions described above. Overall practice was considered to be adequate in only one-third of the ICUs, with no effect of longer experience in using nebulized antibiotics on the rate of adequate practice [71].

This practice pattern challenges the conclusions drawn from some prospective and retrospective studies without standardized nebulization procedures. In many patients, the amount of drug delivered beyond the tip of the endotracheal tube may be negligible, and low drug pulmonary concentrations may enhance the selection of drug-resistant bacteria. The observed practices illustrate the difficulties of implementing optimal nebulization techniques in patients outside of controlled clinical research settings, and this may have implications for future trial designs and the dissemination of knowledge.

## Safety and good practices

In order to guarantee adequate safety and aerosol therapy efficiency during mechanical ventilation, standard operating procedures should be implemented including a checklist for physicians and nurses [34, 72]. Adequate staff training is essential. Key points for good practice with nebulization during mechanical ventilation are summarized in Table 3. Antibiotic nebulization in mechanically ventilated patients is generally well tolerated [6, 34, 69]. Aside from potential toxicities related to systemic absorption, specific nebulization-related side effects need to be considered, however.

**Table 3:** Key good practices for optimal antibiotic nebulization during mechanical ventilation

Organization	Use standard operating procedures and a checklist. Ensure adequate staff training
Nebulizer	Use nebulizers with a small residual volume Do not operate jet nebulizers with gas external to the ventilator
Medication solution	Use solutions for inhalation
Nebulizer position	Position the nebulizer (continuous delivery) upstream in the inspiratory limb at 15–40 cm of the *Y*-piece
Humidification	Remove the heat and moisture exchanger during nebulization; if using a heated humidifier, consider switching it off or use of a dry circuit
Ventilator settings	Volume-controlled constant flow ventilation. Use low respiratory rate, low inspiratory flow and a long inspiratory time
Safety	Place a new filter between the expiratory limb and the ventilator for each nebulization Monitor patients closely during the nebulization, particularly in regard to airway pressure, arterial pressure and oxygen saturation Check for resumption of humidification at the end of the nebulization

### Ventilator dysfunction and circuit obstructions

A filter needs to be positioned between the expiratory limb and the ventilator to protect the latter from expired particles and to prevent dysfunction (Fig. 2). A new filter should be used before each nebulization to prevent progressive obstruction. Mechanical filters appear to be the most effective [73–75]. Obstruction of the expiratory filter is the most serious complication that can arise as it can lead to cardiac arrest [34]. In case of interruption of humidification during the nebulization, its resumption is an important safety condition in order to avoid tracheal tube obstruction.

### Direct mucosal toxicity

Long-term bronchial toxicity and alveolar damage that can result from high local antibiotic concentrations have received scant attention. Whereas a transient benign cough is common, bronchospasm is a more severe, albeit infrequent, side effect that has been reported to occur during antibiotic nebulization [6, 34, 40]. Preventive bronchodilation appears to be unnecessary, although the occurrence of bronchospasm imposes aerosol interruption and bronchodilator nebulization. Tobramycin, colistin and aztreonam are commercially available as solutions for inhalation, whereas amikacin solutions for inhalation, including liposomal forms, are still undergoing investigation [76]. Medications for inhalation should be pyrogen-free, isotonic and sterile, and their pH should be adjusted to that of the airway epithelium (pH 6). Importantly, preservatives and sulfites should be avoided, as they have been specifically associated with adverse effects when inhaled.

### Circuit manipulation and oxygenation

Circuit manipulation for nebulization must follow the usual hygiene standards; the availability of single-patient-use nebulizers contributes to hygiene control. Desaturation and hypoxemia have been reported in patients receiving frequent repeated nebulization [34] due to alveolar derecruitment induced by disconnection of the patient from the ventilator.

### Monitoring

Bronchospasm and obstruction of expiratory filters are first detected as an increase in the peak airway pressure. These complications emphasize the need for close monitoring of the peak airway pressure and oxygenation during nebulization [77]. Systemic absorption of antibiotics may be substantial in patients with renal failure, and drug monitoring is recommended when aminoglycosides are used [36, 37].

## Perspectives

Since 2007, six meta-analyses have been published in regard to nebulized antibiotics as a treatment for lung infections among ventilated patients [60–65], and two meta-analyses evaluated prophylactic nebulized antibiotics [78, 79]. None of them have allowed a definitive conclusion to be reached in regard to possible benefits. As a result, a recent review recommended that use of nebulized antibiotics should be avoided in clinical practice, due to a low level of evidence for their efficacy and the risks of adverse events [80]. Despite this low-quality evidence, recent VAP management guidelines recommend adjunctive nebulized antibiotic therapy for bacteria that are only susceptible to antibiotics when there is evidence for limited efficacy of the intravenous route, i.e., aminoglycosides and colistin. Adjunctive nebulized antibiotic therapy as a treatment of last resort is also recommended [4]. Careful design of future large randomized trials to turn the favorable pharmacokinetic/pharmacodynamic profile of nebulized antibiotics into improved clinical outcomes and reduced toxicity in patients with VAP is needed (Fig. 2). The following considerations should be taken into account in order to comprehensively integrate both technical issues and clinical complexity in a translational research effort.

### Target population

Patients with a high rate of intravenous treatment failure are most susceptible to benefit from this approach. Thus, patients and/or ICUs at high risk of the emergence of MDR bacteria represent target populations for nebulized antibiotics. Defining populations at high risk of toxicity (mainly patients with acute kidney injury) may also be a worthwhile challenge.

### Therapeutic strategy

Intravenous therapy is effective and well tolerated in most patients with VAP caused by β-lactam-susceptible bacteria. The benefit of withholding this therapy is elusive. In patients with late-onset VAP caused by difficult-to-treat bacteria with frequent recurrence, in the light of the questionable benefit of intravenous aminoglycosides, nebulized aminoglycosides as adjunct to systemic therapy may be considered [4, 81]. For the most severely affected patients, who are at very high risk of death, and who are afflicted with pneumonia due to MDR bacteria for which intravenous antibiotics are likely to fail [4, 81], adjunctive high-dose nebulized antibiotics may be beneficial. In patients with VAP due to MDR bacteria that are only susceptible to aminoglycosides or colistin, an exclusive nebulized strategy may be considered. At the other end of the severity spectrum, in patients at risk of developing pneumonia, but who do not yet exhibit parenchymal infection, the benefits of intravenous preemptive antibiotic therapy remain debatable [82–85]. An exclusively nebulized therapeutic strategy may thus warrant evaluation.

### Continuous improvement of nebulization technique

This is urgently needed to implement easy, safe and reproducible techniques as well as to standardize nebulization practices for clinical trials and to thereafter translate the results into clinical practice. A paradigm change may occur in the future with the development of inhaled anti-infective nanoparticle antibody or phage therapies.

## Conclusions

Experimental and clinical pharmacokinetics and pharmacodynamics support the feasibility and possible benefits of nebulized antibiotic therapy to treat VAP in mechanically ventilated patients. Before expanding its clinical use, optimization of nebulization techniques and standardization of nebulization procedures are urgently needed. Large phase III randomized trials are required to demonstrate the clinical effectiveness and benefits in terms of improvements in patient outcomes and reduction in the emergence of antibiotic resistance.

## Abbreviations

CMS: colistimethate sodium
ELF: epithelial lining fluid
ICU: intensive care unit
MDR: multidrug-resistant
MIC: minimum inhibitory concentration
MIU: million international units
VAP: ventilator-associated pneumonia
VAT: ventilator-associated tracheobronchitis

## References

[1] Rodvold KA, George JM, Yoo L (2011). Penetration of anti-infective agents into pulmonary epithelial lining fluid: focus on antibacterial agents. Clin Pharmacokinet.

[2] de Montmollin E, Bouadma L, Gault N, Mourvillier B, Mariotte E, Chemam S (2014). Predictors of insufficient amikacin peak concentration in critically ill patients receiving a 25 mg/kg total body weight regimen. Intensive Care Med.

[3] Kollef MH, Chastre J, Clavel M, Restrepo MI, Michiels B, Kaniga K (2012). A randomized trial of 7-day doripenem versus 10-day imipenem-cilastatin for ventilator-associated pneumonia. Crit Care.

[4] Kalil AC, Metersky ML, Klompas M, Muscedere J, Sweeney DA, Palmer LB (2016). Management of adults with hospital-acquired and ventilator-associated pneumonia: 2016 Clinical practice guidelines by the Infectious Diseases Society of America and the American Thoracic Society. Clin Infect Dis.

[5] Goldstein I, Wallet F, Nicolas-Robin A, Ferrari F, Marquette CH, Rouby JJ (2002). Lung deposition and efficiency of nebulized amikacin during *Escherichia coli* pneumonia in ventilated piglets. Am J Respir Crit Care Med.

[6] Lu Q, Luo R, Bodin L, Yang J, Zahr N, Aubry A (2012). Efficacy of high-dose nebulized colistin in ventilator-associated pneumonia caused by multidrug-resistant *Pseudomonas aeruginosa* and *Acinetobacter baumannii*. Anesthesiology.

[7] Dalhoff A (2014). Pharmacokinetics and pharmacodynamics of aerosolized antibacterial agents in chronically infected cystic fibrosis patients. Clin Microbiol Rev.

[8] Mogayzel PJ, Naureckas ET, Robinson KA, Mueller G, Hadjiliadis D, Hoag JB, Lubsch L, Hazle L, Sabadosa K, Marshall B (2013). Cystic fibrosis pulmonary guidelines. Chronic medications for maintenance of lung health. Am J Respir Crit Care Med.

[9] MacIntyre NR, Silver RM, Miller CW, Schuler F, Coleman RE (1985). Aerosol delivery in intubated, mechanically ventilated patients. Crit Care Med.

[10] Brain JD, Valberg PA (1979). Deposition of aerosol in the respiratory tract. Am Rev Respir Dis.

[11] Ari A, Atalay OT, Harwood R, Sheard MM, Aljamhan EA, Fink JB (2010). Influence of nebulizer type, position, and bias flow on aerosol drug delivery in simulated pediatric and adult lung models during mechanical ventilation. Respir Care.

[12] Harvey CJ, O’Doherty MJ, Page CJ, Thomas SH, Nunan TO, Treacher DF (1997). Comparison of jet and ultrasonic nebulizer pulmonary aerosol deposition during mechanical ventilation. Eur Respir J.

[13] Ferrari F, Liu ZH, Lu Q, Becquemin MH, Louchahi K, Aymard G (2008). Comparison of lung tissue concentrations of nebulized ceftazidime in ventilated piglets: ultrasonic versus vibrating plate nebulizers. Intensive Care Med.

[14] O’Doherty MJ, Thomas SH, Page CJ, Treacher DF, Nunan TO (1992). Delivery of a nebulized aerosol to a lung model during mechanical ventilation. Effect of ventilator settings and nebulizer type, position, and volume of fill. Am Rev Respir Dis.

[15] Wallace SJ, Li J, Rayner CR, Coulthard K, Nation RL (2008). Stability of colistin methanesulfonate in pharmaceutical products and solutions for administration to patients. Antimicrob Agents Chemother.

[16] Boe J, Dennis JH, O’Driscoll BR, Bauer TT, Carone M, Dautzenberg B (2004). Adaptations of the European Respiratory Society guidelines by the Aerosol Therapy Group of the French Lung Society on the use of aerosol therapy through nebulization. Rev Mal Respir.

[17] Dugernier J, Reychler G, Wittebole X, Roeseler J, Depoortere V, Sottiaux T (2016). Aerosol delivery with two ventilation modes during mechanical ventilation: a randomized study. Ann Intensive Care.

[18] Miller DD, Amin MM, Palmer LB, Shah AR, Smaldone GC (2003). Aerosol delivery and modern mechanical ventilation: in vitro/in vivo evaluation. Am J Respir Crit Care Med.

[19] Ehrmann S, Lyazidi A, Louis B, Isabey D, Le Pennec D, Brochard L (2014). Ventilator-integrated jet nebulization systems: tidal volume control and efficiency of synchronization. Respir Care.

[20] Rau JL, Ari A, Restrepo RD (2004). Performance comparison of nebulizer designs: constant-output, breath-enhanced, and dosimetric. Respir Care.

[21] Ari A, Areabi H, Fink JB (2010). Evaluation of aerosol generator devices at 3 locations in humidified and non-humidified circuits during adult mechanical ventilation. Respir Care.

[22] Boukhettala N, Poree T, Diot P, Vecellio L (2015). In vitro performance of spacers for aerosol delivery during adult mechanical ventilation. J Aerosol Med Pulm Drug Deliv.

[23] Lin HL, Fink JB, Zhou Y, Cheng YS (2009). Influence of moisture accumulation in inline spacer on delivery of aerosol using metered-dose inhaler during mechanical ventilation. Respir Care.

[24] Villafane MC, Cinnella G, Lofaso F, Isabey D, Harf A, Lemaire F (1996). Gradual reduction of endotracheal tube diameter during mechanical ventilation via different humidification devices. Anesthesiology.

[25] Dhand R (2003). Maximizing aerosol delivery during mechanical ventilation: go with the flow and go slow. Intensive Care Med.

[26] Dhand R (2000). Special problems in aerosol delivery: artificial airways. Respir Care.

[27] Dugernier J, Wittebole X, Roeseler J, Michotte JB, Sottiaux T, Dugernier T (2015). Influence of inspiratory flow pattern and nebulizer position on aerosol delivery with a vibrating-mesh nebulizer during invasive mechanical ventilation: an in vitro analysis. J Aerosol Med Pulm Drug Deliv.

[28] Carcas AJ, Garcia-Satue JL, Zapater P, Frias-Iniesta J (1999). Tobramycin penetration into epithelial lining fluid of patients with pneumonia. Clin Pharmacol Ther.

[29] Taccone FS, Laterre PF, Spapen H, Dugernier T, Delattre I, Layeux B (2010). Revisiting the loading dose of amikacin for patients with severe sepsis and septic shock. Crit Care.

[30] Lu Q, Girardi C, Zhang M, Bouhemad B, Louchahi K, Petitjean O (2010). Nebulized and intravenous colistin in experimental pneumonia caused by *Pseudomonas aeruginosa*. Intensive Care Med.

[31] Imberti R, Cusato M, Villani P, Carnevale L, Iotti GA, Langer M (2010). Steady-state pharmacokinetics and BAL concentration of colistin in critically ill patients after IV colistin methanesulfonate administration. Chest.

[32] Markou N, Fousteri M, Markantonis SL, Boutzouka E, Tsigou E, Baltopoulo G (2011). Colistin penetration in the alveolar lining fluid of critically ill patients treated with IV colistimethate sodium. Chest.

[33] Ferrari F, Lu Q, Girardi C, Petitjean O, Marquette CH, Wallet F (2009). Nebulized ceftazidime in experimental pneumonia caused by partially resistant *Pseudomonas aeruginosa*. Intensive Care Med.

[34] Lu Q, Yang J, Liu Z, Gutierrez C, Aymard G, Rouby JJ (2011). Nebulized ceftazidime and amikacin in ventilator-associated pneumonia caused by *Pseudomonas aeruginosa*. Am J Respir Crit Care Med.

[35] Paul M, Lador A, Grozinsky-Glasberg S, Leibovici L (2014). Beta lactam antibiotic monotherapy versus beta lactam-aminoglycoside antibiotic combination therapy for sepsis. Cochrane Database Syst Rev.

[36] Badia JR, Soy D, Adrover M, Ferrer M, Sarasa M, Alarcon A (2004). Disposition of instilled versus nebulized tobramycine and imipenem in ventilated intensive care unit (ICU) patients. J Antimicrob Chemother.

[37] Petitcollin A, Dequin PF, Darrouzain F, Vecellio L, Boulain T, Garot D (2016). Pharmacokinetics of high-dose nebulized amikacin in ventilated critically ill patients. J Antimicrob Chemother.

[38] Ferrari F, Goldstein I, Nieszkowszka A, Elman M, Marquette CH, Rouby JJ (2003). Lack of lung tissue and systemic accumulation after consecutive daily aerosols of amikacin in ventilated piglets with healthy lungs. Anesthesiology.

[39] Landersdorfer CB, Nguyen TH, Lieu LT, Nguyen G, Bischof RJ, Meeusen EN (2017). Substantial targeting advantage achieved by pulmonary administration of colistin methanesulfonate in a large-animal model. Antimicrob Agents Chemother.

[40] Luyt CE, Clavel M, Guntupalli K, Johannigman J, Kennedy JI, Wood C (2009). Pharmacokinetics and lung delivery of PDDS-aerosolized amikacin (NKTR-061) in intubated and mechanically ventilated patients with nosocomial pneumonia. Crit Care.

[41] Niederman MS, Chastre J, Corkery K, Fink JB, Luyt CE, Garcia MS (2012). BAY41-6551 achieves bactericidal tracheal aspirate amikacin concentrations in mechanically ventilated patients with Gram-negative pneumonia. Intensive Care Med.

[42] Dhand R, Sohal H (2008). Pulmonary drug delivery system for inhalation therapy in mechanically ventilated patients. Expert Rev Med Devices.

[43] Montgomery AB, Vallance S, Abuan T, Tservistas M, Davies A (2014). A randomized double-blind placebo-controlled dose-escalation phase 1 study of aerosolized amikacin and fosfomycin delivered via the PARI investigational eFlow(R) inline nebulizer system in mechanically ventilated patients. J Aerosol Med Pulm Drug Deliv.

[44] Athanassa ZE, Markantonis SL, Fousteri MZ, Myrianthefs PM, Boutzouka EG, Tsakris A (2012). Pharmacokinetics of inhaled colistimethate sodium (CMS) in mechanically ventilated critically ill patients. Intensive Care Med.

[45] Boisson M, Jacobs M, Gregoire N, Gobin P, Marchand S, Couet W (2014). Comparison of intrapulmonary and systemic pharmacokinetics of colistin methanesulfonate (CMS) and colistin after aerosol delivery and intravenous administration of CMS in critically ill patients. Antimicrob Agents Chemother.

[46] Bihan K, Lu Q, Enjalbert M, Apparuit M, Langeron O, Rouby JJ (2016). Determination of colistin and colistimethate levels in human plasma and urine by high-performance liquid chromatography-tandem mass spectrometry. Ther Drug Monit.

[47] Sorli L, Luque S, Grau S, Berenguer N, Segura C, Montero MM (2013). Trough colistin plasma level is an independent risk factor for nephrotoxicity: a prospective observational cohort study. BMC Infect Dis.

[48] Kiem S, Schentag JJ (2014). Interpretation of epithelial lining fluid concentrations of antibiotics against methicillin resistant staphylococcus aureus. Infect Chemother.

[49] Mukker JK, Singh RS, Derendorf H (2015). Pharmacokinetic and pharmacodynamic implications in inhalable antimicrobial therapy. Adv Drug Deliv Rev.

[50] Palmer LB, Smaldone GC, Chen JJ, Baram D, Duan T, Monteforte M (2008). Aerosolized antibiotics and ventilator-associated tracheobronchitis in the intensive care unit. Crit Care Med.

[51] Palmer LB, Smaldone GC (2014). Reduction of bacterial resistance with inhaled antibiotics in the intensive care unit. Am J Respir Crit Care Med.

[52] Kollef MH, Ricard JD, Roux D, Francois B, Ischaki E, Rozgonyi Z (2016). A randomized trial of the amikacin fosfomycin inhalation system for the adjunctive therapy of Gram-negative ventilator-associated pneumonia: IASIS Trial. Chest.

[53] Rattanaumpawan P, Lorsutthitham J, Ungprasert P, Angkasekwinai N, Thamlikitkul V (2010). Randomized controlled trial of nebulized colistimethate sodium as adjunctive therapy of ventilator-associated pneumonia caused by Gram-negative bacteria. J Antimicrob Chemother.

[54] Abdellatif S, Trifi A, Daly F, Mahjoub K, Nasri R, Ben Lakhal S (2016). Efficacy and toxicity of aerosolised colistin in ventilator-associated pneumonia: a prospective, randomised trial. Ann Intensive Care.

[55] Kofteridis DP, Alexopoulou C, Valachis A, Maraki S, Dimopoulou D, Georgopoulos D (2010). Aerosolized plus intravenous colistin versus intravenous colistin alone for the treatment of ventilator-associated pneumonia: a matched case-control study. Clin Infect Dis.

[56] Korbila IP, Michalopoulos A, Rafailidis PI, Nikita D, Samonis G, Falagas ME (2010). Inhaled colistin as adjunctive therapy to intravenous colistin for the treatment of microbiologically documented ventilator-associated pneumonia: a comparative cohort study. Clin Microbiol Infect.

[57] Tumbarello M, De Pascale G, Trecarichi EM, De Martino S, Bello G, Maviglia R (2013). Effect of aerosolized colistin as adjunctive treatment on the outcomes of microbiologically documented ventilator-associated pneumonia caused by colistin-only susceptible gram-negative bacteria. Chest.

[58] Dalfino L, Puntillo F, Mosca A, Monno R, Spada ML, Coppolecchia S (2012). High-dose, extended-interval colistin administration in critically ill patients: is this the right dosing strategy? A preliminary study. Clin Infect Dis.

[59] Poudyal A, Howden BP, Bell JM, Gao W, Owen RJ, Turnidge JD (2008). In vitro pharmacodynamics of colistin against multidrug-resistant *Klebsiella pneumoniae*. J Antimicrob Chemother.

[60] Florescu DF, Qiu F, McCartan MA, Mindru C, Fey PD, Kalil AC (2012). What is the efficacy and safety of colistin for the treatment of ventilator-associated pneumonia? A systematic review and meta-regression. Clin Infect Dis.

[61] Ioannidou E, Siempos II, Falagas ME (2007). Administration of antimicrobials via the respiratory tract for the treatment of patients with nosocomial pneumonia: a meta-analysis. J Antimicrob Chemother.

[62] Valachis A, Samonis G, Kofteridis DP (2015). The role of aerosolized colistin in the treatment of ventilator-associated pneumonia: a systematic review and metaanalysis. Crit Care Med.

[63] Zampieri FG, Nassar AP, Gusmao-Flores D, Taniguchi LU, Torres A, Ranzani OT (2015). Nebulized antibiotics for ventilator-associated pneumonia: a systematic review and meta-analysis. Crit Care.

[64] Russell CJ, Shiroishi MS, Siantz E, Wu BW, Patino CM (2016). The use of inhaled antibiotic therapy in the treatment of ventilator-associated pneumonia and tracheobronchitis: a systematic review. BMC Pulm Med.

[65] Sole-Lleonart C, Rouby JJ, Blot S, Poulakou G, Chastre J, Palmer LB (2017). Nebulization of antiinfective agents in invasively mechanically ventilated adults: a systematic review and meta-analysis. Anesthesiology.

[66] Karvouniaris M, Makris D, Zygoulis P, Triantaris A, Xitsas S, Mantzarlis K (2015). Nebulised colistin for ventilator-associated pneumonia prevention. Eur Respir J.

[67] Wood GC, Boucher BA, Croce MA, Hanes SD, Herring VL, Fabian TC (2002). Aerosolized ceftazidime for prevention of ventilator-associated pneumonia and drug effects on the proinflammatory response in critically ill trauma patients. Pharmacotherapy.

[68] Ehrmann S, Roche-Campo F, Sferrazza Papa GF, Isabey D, Brochard L, Apiou-Sbirlea G (2013). Aerosol therapy during mechanical ventilation: an international survey. Intensive Care Med.

[69] Ehrmann S, Roche-Campo F, Bodet-Contentin L, Razazi K, Dugernier J, Trenado-Alvarez J (2016). Aerosol therapy in intensive and intermediate care units: prospective observation of 2808 critically ill patients. Intensive Care Med.

[70] Sole-Lleonart C, Roberts JA, Chastre J, Poulakou G, Palmer LB, Blot S (2016). Global survey on nebulization of antimicrobial agents in mechanically ventilated patients: a call for international guidelines. Clin Microbiol Infect.

[71] Sole-Lleonart C, Rouby JJ, Chastre J, Poulakou G, Palmer LB, Blot S (2016). Intratracheal administration of antimicrobial agents in mechanically ventilated adults: an international survey on delivery practices and safety. Respir Care.

[72] Rello J, Rouby JJ, Sole-Lleonart C, Chastre J, Blot S, Luyt CE (2017). Key conceptional considerations on nebulization of antimicrobial agents to mechanically ventilated patients. Clin Microbiol Infect.

[73] Mostofi R, Wang B, Haghighat F, Bahloul A, Jaime L (2010). Performance of mechanical filters and respirators for capturing nanoparticles—limitations and future direction. Ind Health.

[74] Rengasamy S, BerryAnn R, Szalajda J (2013). Nanoparticle filtration performance of filtering facepiece respirators and canister/cartridge filters. J Occup Environ Hyg.

[75] Tonnelier A, Lellouche F, Bouchard PA, L’Her E (2013). Impact of humidification and nebulization during expiratory limb protection: an experimental bench study. Respir Care.

[76] Antoniu S, Azoicai D (2013). Novel amikacin inhaled formulation for the treatment of lower respiratory tract infections. Drugs Today (Barc).

[77] Rouby JJ, Bouhemad B, Monsel A, Brisson H, Arbelot C, Lu Q (2012). Aerosolized antibiotics for ventilator-associated pneumonia: lessons from experimental studies. Anesthesiology.

[78] Falagas ME, Siempos II, Bliziotis IA, Michalopoulos A (2006). Administration of antibiotics via the respiratory tract for the prevention of ICU-acquired pneumonia: a meta-analysis of comparative trials. Crit Care.

[79] Roquilly A, Marret E, Abraham E, Asehnoune K (2015). Pneumonia prevention to decrease mortality in intensive care unit: a systematic review and meta-analysis. Clin Infect Dis.

[80] Rello J, Solé-Lleonart C, Rouby JJ, Chastre J, Blot S, Poulakou G (2017). Use of nebulized antimicrobials for the treatment of respiratory infections in invasively mechanically ventilated adults: a position paper from the european society of clinical microbiology and infectious diseases. Clin Microbiol Infect.

[81] American Thoracic Society (2005). Guidelines for the management of adults with hospital-acquired, ventilator-associated, and healthcare-associated pneumonia. Am J Respir Crit Care Med.

[82] Hurley JC (2014). Topical antibiotics as a major contextual hazard toward bacteremia within selective digestive decontamination studies: a meta-analysis. BMC Infect Dis.

[83] Nseir S, Di Pompeo C, Pronnier P, Beague S, Onimus T, Saulnier F (2002). Nosocomial tracheobronchitis in mechanically ventilated patients: incidence, aetiology and outcome. Eur Respir J.

[84] Nseir S, Martin-Loeches I, Makris D, Jaillette E, Karvouniaris M, Valles J (2014). Impact of appropriate antimicrobial treatment on transition from ventilator-associated tracheobronchitis to ventilator-associated pneumonia. Crit Care.

[85] Vincent JL, Jacobs F (2011). Effect of selective decontamination on antibiotic resistance. Lancet Infect Dis.

